# Copper-Lithium-Doped Nanohydroxyapatite Modulates Mesenchymal Stem Cells Homing to Treat Glucocorticoids-Related Osteonecrosis of the Femoral Head

**DOI:** 10.3389/fbioe.2022.916562

**Published:** 2022-06-02

**Authors:** Qianhao Li, Zhouyuan Yang, Zhun Wei, Donghai Li, Yue Luo, Pengde Kang

**Affiliations:** Orthopedic Research Institute, Department of Orthopedics, West China Hospital, Sichuan University, Chengdu, China

**Keywords:** copper-lithium-doped nanohydroxyapatite, osteonecrosis of the femoral head, mesenchymal stem cells, cell homing, *in situ* tissue regeneration

## Abstract

*In situ* tissue regeneration has been demonstrated to promote bone repair. To identify a better approach for treating osteonecrosis of the femoral head (ONFH), we prepared scaffolds using copper-lithium-doped nanohydroxyapatite (Cu-Li-nHA), which has the potential to modulate mesenchymal stem cells (MSCs) homing. The scaffold was fabricated using the gas foaming method and the migration, angiogenesis, and osteogenesis activities of MSCs were detected using Transwell assays, tube formation assays, alkaline phosphatase and alizarin red S staining, respectively. We then implanted the Cu-Li-nHA scaffold into the femoral heads of ONFH rabbits, and CFSE labeled exogenous MSCs were injected intravenously to verify cell homing. The repair effect was subsequently examined using micro-CT and histological analysis *in vivo*. The results showed that Cu-Li-nHA significantly promoted MSCs migration and homing by upregulating the HIF-1α/SDF-1 pathway. The Cu-Li-nHA group showed optimal osteogenesis and angiogenesis and greater improvements in new bone formation in ONFH rabbits. To summarize, Cu-Li-nHA promoted homing and induced the osteogenic differentiation of MSCs, thereby enhancing bone regeneration during ONFH repair. Thus, Cu-Li-nHA implantation may serve as a potential therapeutic strategy for ONFH in the future.

## Introduction

The excessive long-term use of glucocorticoids (GCs) is one of the major etiologies of osteonecrosis of the femoral head (ONFH), a condition that decreases a patient’s quality of life (Chughtai et al., 2017; Mont et al., 2020). Moreover, most patients who experience a collapse of the femoral head need to undergo total hip arthroplasty (THA) (Moya-Angeler et al., 2015). Unfortunately, the mechanisms underlying GCs-ONFH remain unclear, although, abnormal differentiation of bone marrow stem cells (BMSCs), apoptosis of osteocytes, and tissue ischemia may play important roles in its pathogenesis (Zhun et al., 2018; Chang et al., 2020). Studies have demonstrated that BMSCs derived from patients with GCs-ONFH exhibit poorer osteogenic differentiation activity than those derived from healthy individuals (Houdek et al., 2016). High-dose GCs can inhibit the Wnt/β-catenin pathway by upregulating the expression of DKK-1, which prevents the differentiation of BMSCs into osteoblasts and induces adipogenesis (Kato et al., 2018; Zhun et al., 2018). In recent years, biomaterials that promote osteogenesis have made great progress in the treatment of ONFH (Zhao and Ma, 2020; Zhu et al., 2020; Zhu et al., 2022).

Nanohydroxyapatite (nHA) has been widely used in clinical applications, especially for bone defect repair, owing to its excellent biocompatibility and osteoconductivity (Winkler et al., 2018). However, their insufficient osteoinductivity limits the use. Considerable efforts have been made to improve the osteogenesis of nHA by doping metal ions (such as strontium and lithium) into it, which could effectively contribute to the osteoblast differentiation of BMSCs (Ge et al., 2018; Li et al., 2018a). Lithium (Li), a common psychotropic drug, has been shown to enhance bone regeneration by activating the Wnt/GSK-3β pathway (Li et al., 2011). Lithium-doped nanohydroxyapatite (Li-nHA) scaffolds have better osteoinductivity than nHA for repairing bone defects (Li et al., 2018a). However, although Li-nHA improves the cell adherence and differentiation of BMSCs, it cannot help recruit more BMSCs to necrotic areas. Mesenchymal stem cells (MSCs) therapies have been widely studied for the treatment of various diseases (Wang Z. et al., 2022). Although researchers have implanted BMSCs into the femoral head to improve osteogenesis, the complex process of implantation and low survival rate of BMSCs limit the extensive use of this method (Kang et al., 2018; Zhou et al., 2021). An approach known as *in situ* tissue regeneration has been introduced, which can mobilize host endogenous stem cells to target tissues (Ko et al., 2013). Therefore, a scaffold that can modulate the host microenvironment to recruit MSCs to the damaged regions of ONFH is required.

**FIGURE 1:**
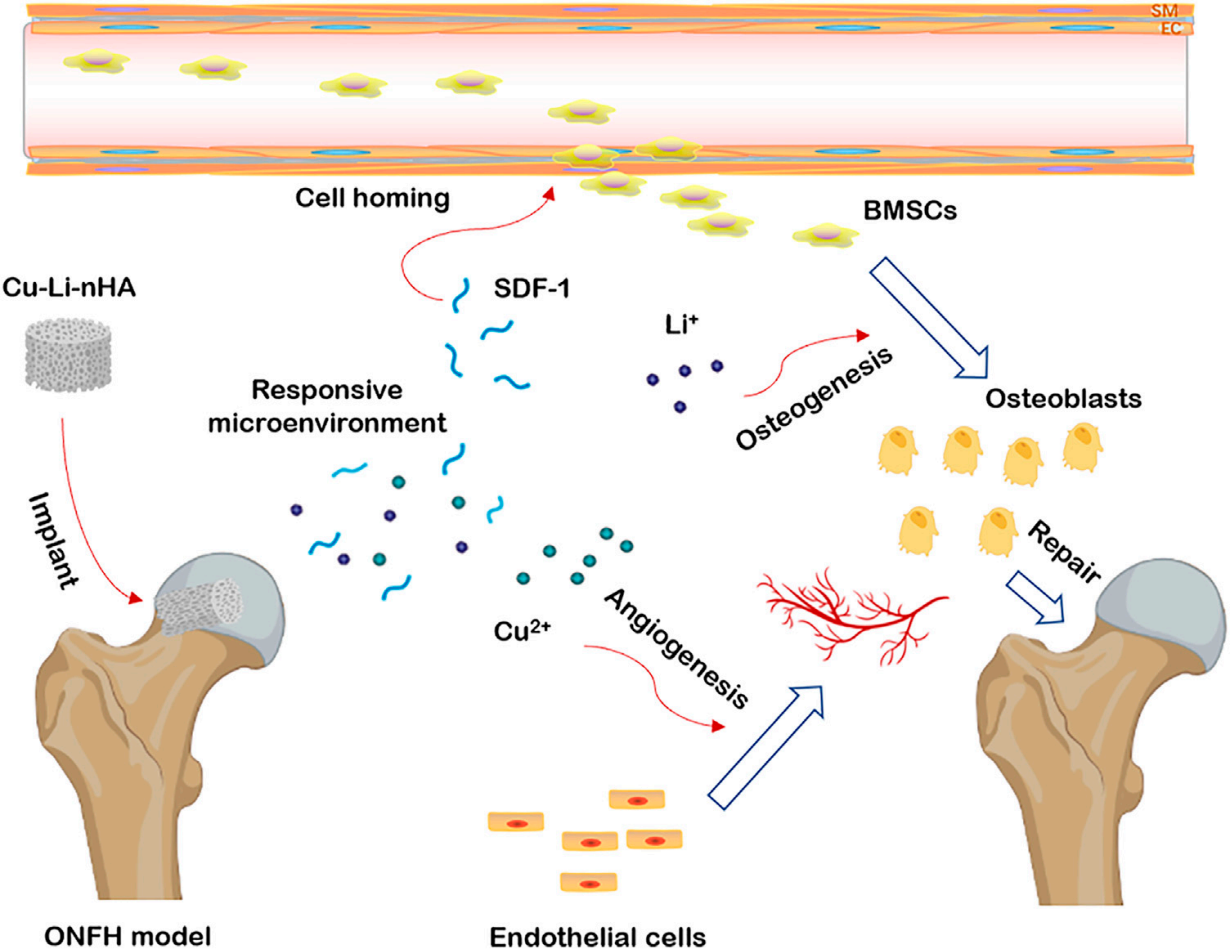
The graphical abstract of the study. Cu-Li-nHA implantation could stimulate adjacent tissue and build an optimal microenvironment *via* releasing Li^+^ and Cu^2+^, and promote cell homing, osteogenesis and angiogenesis to repair ONFH.

Copper (Cu) ions can stabilize the expression of hypoxia-inducible factor-1α (HIF-1α) and upregulate vascular endothelial growth factor (VEGF) to induce neovascularization (Feng et al., 2009; Li et al., 2021). Angiogenesis also promotes bone development (Hu and Olsen, 2016; Wang L. et al., 2022). After co-culture with BMSCs, Cu-containing bioactive glass scaffolds increased the expression of VEGF and osteocalcin (OCN) simultaneously (Wu et al., 2013). Interestingly, studies have indicated that Cu has the potential to induce cell homing (Meng et al., 2015). After implanting Cu-containing microbubbles in the ischemic infarct area of the heart, BMSCs migrate to this area for regeneration. Cu may elevate the levels of localized stromal cell-derived factor-1 (SDF-1), thus, leading to the recruitment of BMSCs *via* chemotactic attraction (Lau and Wang, 2011; Zhu et al., 2018). Here, according to the *in situ* tissue regeneration concept, we have developed a copper-lithium-doped nanohydroxyapatite (Cu-Li-nHA) composite scaffold that releases Cu ions by upregulating SDF-1 expression, promotes MSCs homing to the necrotic zone, and induces the differentiation of recruited MSCs *via* osteoblastogenesis through Wnt/β-catenin signaling activation. Finally, these synergistic effects contribute to bone repair in patients with ONFH (Figure 1).

## Materials and Methods

### Synthesis and Characterization of Cu-Li-nHA

Cu-nHA and Li-nHA were synthesized by liquid-phase coprecipitation and were mixed to prepare Cu-Li-nHA using the gas foaming method. The doped copper had a molar ratio of 0.25%, and a lithium content of 1.5% (Li et al., 2018b; Li et al., 2021). The synthesis was performed as follows: 1) (NH_4_)_2_HPO_4_ (Sinopharm, China) solution was added dropwise to the Ca(NO_3_)_2_·4H_2_O (Sinopharm, China) solution containing LiNO_3_ or Cu(NO_3_)_2_ (Sigma, United States), and then colloidal fluid was separated by a standing and layering process and the precipitate was rinsed and sintered at 1,000°C for 2 h in a muffle furnace to obtain Cu-nHA and Li-nHA powders; 2) 0.25% Cu-nHA and 1.5% Li-nHA powders (1:1 mass) were mixed using a planetary ball mill with a rotating speed of 45 Hz for 10 min to obtain Cu-Li-nHA powders; and 3) The slurry was prepared using a mixture of 5% polyvinyl alcohol, followed by the addition of 10 ml of H_2_O_2_, and the mixture was then stirred and heated repeatedly until a slurry was filled with foam. Finally, the slurry was dried and the scaffold precursors were sintered at 1,150°C for 4 h to form porous scaffolds.

The materials were characterized using X-ray diffraction (XRD, Shimadzu, Japan) and Fourier-transform infrared spectroscopy (FTIR, Thermo, United States). Transmission electron microscopy (TEM, FEI, United States) was used to observe the particle size, and scanning electron microscopy (SEM, JEOL, Japan) was used to observe the scaffold structure. The porosity was analyzed using mercury intrusion (Micromeritics, United States), and the compressive performance was measured using an electronic universal testing machine (Shimadzu, Japan).

### Effects of Cu-Li-nHA on the Differentiation and Migration of BMSCs

BMSCs were isolated from newborn New Zealand white rabbit pups and identified using a previously published method (shown in the Supplementary Material) (Li et al., 2018a). BMSCs were co-cultured with Cu-Li-nHA, Li-nHA and nHA (Cu-Li-nHA + BMSCs, Li-nHA + BMSCs, nHA + BMSCs) in an incubator at 37°C, 5% CO_2_ for 7 days. Cell adhesion was observed using SEM. In addition, BMSCs were inoculated into 6-well plates containing extract solutions of different materials per well at a density of 5×10^4^/ml. Alkaline phosphatase and alizarin red S staining assays were performed to test the osteogenic differentiation of BMSCs at 2 weeks.

Tube formation assays were performed to evaluate neovascularization. Starvation-treated human umbilical vein endothelial cells were inoculated in 48-well plates at a density of 2×10^4^/ml. The groups were set as follows: 1) Cu-Li-nHA group: Cu-Li-nHA + BMSCs co-culture medium, 2) Li-nHA group: Li-nHA + BMSCs co-culture medium, 3) nHA group: nHA + BMSCs co-culture medium, 4) positive group: complete medium +50 ng/ml recombinant VEGF protein (SinoBiological, China), and 5) negative group: complete medium. Closed lumens were observed using an inverted microscope (Olympus, Japan).

Transwell assays were performed to detect migration of BMSCs. BMSCs and scaffolds were co-cultured for 7 days to prepare the conditioned medium. Conditioned or complete medium was added to the lower chamber of a 24-well Transwell plate for the following five groups: 1) Cu-Li-nHA, 2) Li-nHA, 3) nHA, 4) SDF-1 (complete medium +100 ng/ml recombinant protein of SDF-1) (SinoBiological, China), and 5) negative (complete medium). One milliliter of the CFSE-labeled BMSCs suspension (5×10^4^/ml) was dropped into the upper chamber. Transwell plates were incubated for 8 h in an incubator at 37°C and 5% CO_2_ and were finally observed by fluorescence microscopy (Olympus, Japan).

The expression levels of Runx2, β-catenin, HIF-1α, VEGF, and SDF-1 were verified using reverse transcription-polymerase chain reaction (RT-PCR) and western blotting. 1) RT-PCR: RNA was isolated from BMSCs using TRIzol reagent (Invitrogen, United States). Primer sequences for each gene are shown in Table 1. PCR amplification was performed using real-time PCR (QuantStudio 3, ABI, United States). The pre-reaction was at 95°C for 10 min, and 40 reaction cycles were performed. The parameters were set as follows: 95°C for 15 s, 55°C for 30 s, and 72°C for 30 s. 2) Western blotting: The total protein concentration was determined using the BCA method (Epizyme, China). Proteins were separated by electrophoresis (BioRad, United States) and then diluted primary antibodies (1:1,000) were added and the membranes were incubated at 4°C overnight. The membranes were then incubated with secondary antibodies (1:5,000) at 37°C for 60 min. The bands were obtained using an imaging system (BioRad, United States).

**TABLE 1:** RNA primer sequence.

RNA	Primer sequence
RUNX2	F: 5′GGA​CGA​GGC​AAG​AGT​TTC​ACT​T3′
	R: 5′CTG​TCT​GTG​CCT​TCT​TGG​TTC​C3′
β-catenin	F: 5′TCT​GCT​ATT​GTA​CGC​ACC​AT3′
	R: 5′CTG​CCA​TTT​TAG​CTC​CTT​CT3′
HIF-1α	F: 5′TCG​AAG​TAG​TGC​TGA​CCC​TG3′
	R: 5′ACT​GGT​AGG​CTC​AGG​TGA​AC3′
VEGF	F: 5′TCT​ACC​TCC​ACC​ATG​CCA​AG3′
	R: 5′CAC​GCA​CTC​CAG​GCT​TTC​AT3′
SDF-1	F: 5′GCT​CTG​CAT​CAG​TGA​CGG​TA3′
	R: 5′TAA​TTT​CGG​GTC​AA-TGC​ACA3′
β-actin	F: 5′CGT​CTT​CCC​CTC​CAT​CGT​G3′
	R: 5′GGG​TAC​TTG​AGC​GTC​AGG​AT3′

### Establishment of the ONFH Model and Material Implantations

The animal study protocol was reviewed and approved by the Animal Care and Use Committee of Sichuan University (2020091A). Thirty New Zealand white rabbits (male, aged 28–32 weeks, weight 2.5–3.2 kg, one per cage) were housed at the animal center of our institution and maintained on a standard laboratory diet and water. The rabbit model of ONFH was established by intramuscular injection of lipopolysaccharide (10 μg/kg, Sigma, United States) combined with intramuscular injection of methylprednisolone (20 mg/kg, Sigma, United States) for the following 3 days (Li et al., 2022).

All animals were labeled with an implantable RFID chip tag and then randomly grouped (six for each) using SPSS software as follows: 1) nHA group, 2) Li-nHA group, 3) Cu-Li-nHA group, 4) negative group (only surgically drilled), and 5) blank group. Drilling and material implantation were performed using the posterior-lateral approach 2 weeks after ONFH modeling in groups 1–4. Materials were implanted after drilling a tunnel (diameter, 3.5 mm) below the junction of the femoral head and neck and 1 cm in depth along the axial direction of the femoral neck (Supplementary Figures S4, S5).

### *In Vivo* Evaluation of BMSCs Homing by Cu-Li-nHA

Four weeks after surgery, all groups except for group 4 were injected with 1 ml of exogenous BMSCs (5×10^6^/ml) labeled with fluorescent CFSE *via* ear margin veins. Two weeks later, two rabbits in each group were sacrificed to perform immunofluorescence assays to verify cell homing.

Femoral head samples were obtained at 6 and 12 weeks after surgery. The following tests were performed to detect implanted materials and new bone reconstruction: 1) Micro-CT and bone volume (BV)/total volume (TV) analysis; 2) HE and Goldner staining; and 3) immunohistochemistry of OCN, Runx2, GSK-3β, β-catenin, VEGF, and SDF-1 (Abcam, United States).

After fixing with 10% neutral formaldehyde for 7 days, the samples were scanned by micro-CT (Quantum GX II, Perkin Elmer, United States) at a voltage of 90 kV, a current of 88 A, and a voxel size of 50 μm. Then, the samples were decalcified in 20% EDTA for 14 days and then embedded in paraffin. Coronally sections were sliced at a thickness of 3 μm, dewaxed in xylene, and hydrated with graded ethanol series before staining with the HE reagent kit (Beyotime, China). To analyze the area of mineralized bone, Goldner staining was performed sequentially following the protocols of the stain kit (Solarbio, China) using Weigert iron hematoxylin solution, Ponceau solution, Orange G solution, and Light Green solution. The sections for detecting cell homing were incubated with the diluted primary antibody of Collagen I (1:200), and then labeled with the secondary antibody of Cy5. DAPI (1 μg/ml) counterstained sections were observed under the confocal microscope (Nikon, Japan). Immunohistochemistry was conducted with diluted primary antibodies (1:200) at 4°C overnight and visualized with a secondary antibody. Immunohistochemical proteins were analyzed using the positive staining area ratio.

### Statistical Analysis

For the semi-quantitative analysis above, three researchers blinded to the group allocation used ImageJ software (National Institutes of Health, United States) to analyze each sample, and the average results were determined after three repeated measurements.

SPSS 22.0 (SPSS Inc., Chicago) was used to perform statistical analysis, and significant differences were defined at *p* < 0.05. All continuous variables are expressed as the mean ± standard deviation (mean ± SD). The results were analyzed using a one-way analysis of variance with post-hoc Bonferroni’s multiple comparisons test.

## Results

### Characterization of Cu-Li-nHA

**FIGURE 2:**
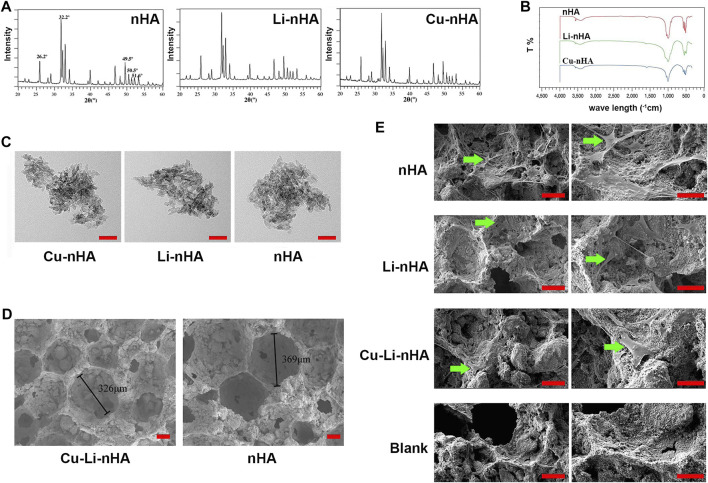
**(A)** XRD spectrum. **(B)** FITR spectrum. **(C)** TEM observation for particle size. Scale bar in the bottom right-hand corner of each image (red): 50 nm. **(D)** SEM observation for porous scaffold structure. Pore size is shown in each image. Scale bar in the bottom right-hand corner of each image (red): 100 μm. **(E)** SEM observation for BMSCs adhesion to scaffolds. Blank Group: nHA without BMSCs co-culture. Green arrow: adherent BMSCs. Scale bar in the bottom right-hand corner of each image in the left column (red): 100 μm; Scale bar in the bottom right-hand corner of each image in the right column (red): 50 μm.

The characteristic peaks of Li-nHA or Cu-nHA were detected at 26.2°, 32.2°, 49.5°, 50.5° and 51.6°, and these findings are consistent with the spectrum of the standard nHA (Figure 2A). The FTIR results also showed a similar spectrum for the three groups. The peak at the wave length near 3,500 cm^−1^ was the absorption peak of hydroxyl groups (O-H), while the peaks at the wave length near 1,000 cm^−1^ and 600 to 500 cm^−1^ were the absorption peaks of phosphate (PO_4_^3−^) (Figure 2B). TEM showed that each particle was composed of several needle- or rod-like structures with long diameter less than 50 nm, thus, confirming the sizes of Cu-nHA and Li-nHA were at the nanometer level (Figure 2C). The SEM analysis showed that the gas foaming method could prepare a Cu-Li-nHA three-dimensional porous scaffold with see-through pores between macropores and the interconnections of pores (Figure 2D). Cu-Li-nHA and nHA had no significant differences in porosity (74.65 ± 11.33% vs. 71.59 ± 11.09%, *p* > 0.05) or compressive strength (4.79 ± 0.92 MPa vs. 4.82 ± 0.85 MPa, *p* > 0.05).

### Cu-Li-nHA Promoted BMSCs Migration, Osteogenesis, and Angiogenesis

**FIGURE 3:**
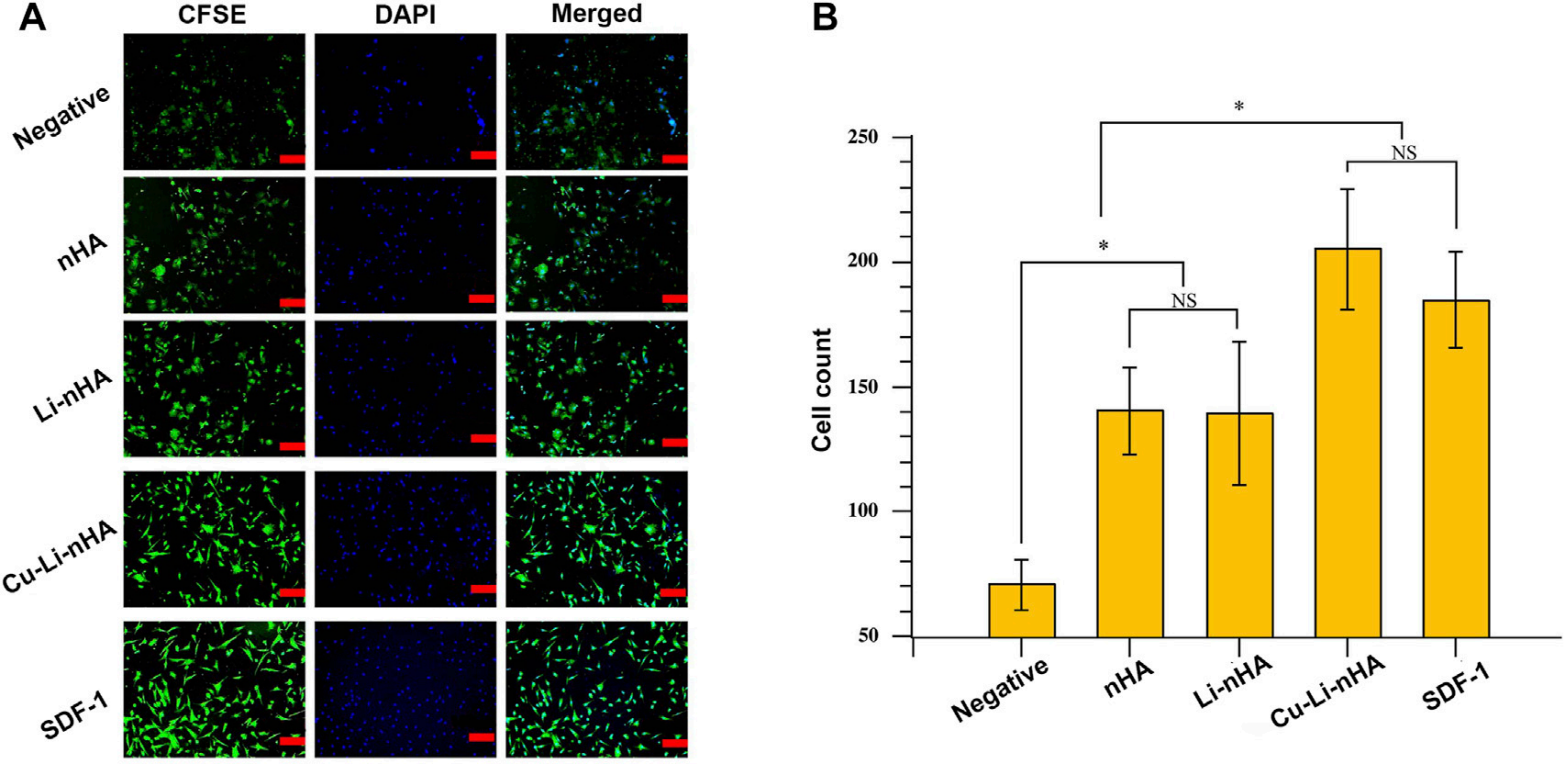
**(A)** Immunofluorescence assays of BMSCs migration using Transwell device. BMSCs were labeled by CFSE (green) and DAPI (blue). Scale bar in the bottom right-hand corner of each image (red): 100 μm. **(B)** The number of migrated cells under the membrane among different groups. Negative Group: complete medium; SDF-1 Group: SDF-1 + complete medium. Statistical analysis was conducted using a one-way analysis of variance with post-hoc Bonferroni’s multiple comparisons test. NS: *p* > 0.05, *p < 0.05.

**FIGURE 4:**
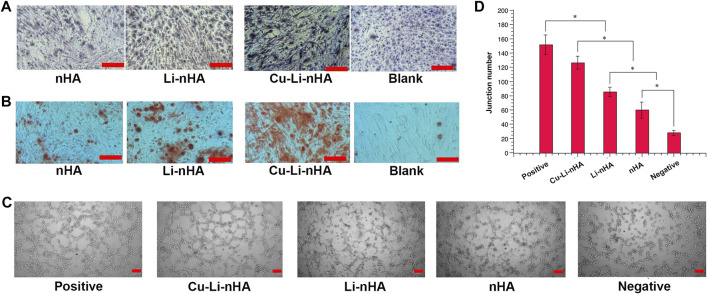
**(A,B)** Osteogenic differentiation tests. **(A)** Alkaline phosphatase staining among groups. Light or dark purple staining cells represent positive cells. **(B)** Alizarin red S staining among groups. The red areas or dots represent calcium nodules or deposits. Blank Group: complete medium. Scale bar in the bottom right-hand corner of each image (red): 200 μm. **(C,D)** Tube formation assays for angiogenesis test. **(C)** Tube-like structure represents newly formed vessel. Scale bar in the bottom right-hand corner of each image (red): 200 μm. **(D)** The number of tube-like structures among different groups. Positive Group: VEGF + complete medium; Negative Group: complete medium. Statistical analysis was conducted using a one-way analysis of variance with post-hoc Bonferroni’s multiple comparisons test. *p < 0.05.

**FIGURE 5:**
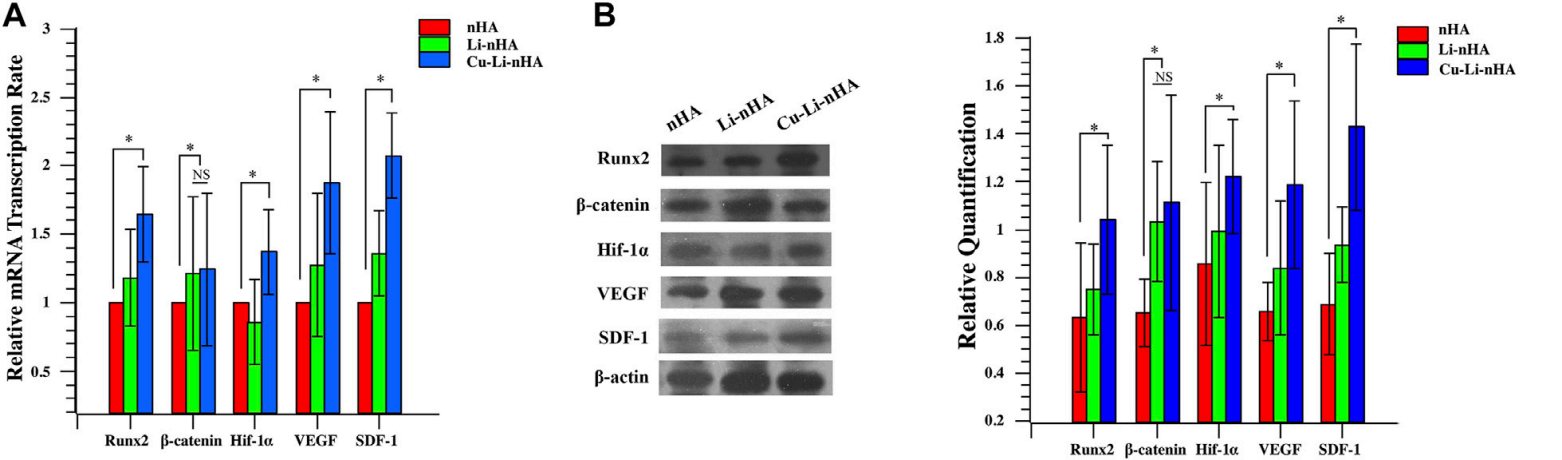
**(A)** RT-PCR and **(B)** Western-blotting of RUNX2, β-catenin, HIF-1α, VEGF and SDF-1 in BMSCs. Statistical analysis was conducted using a one-way analysis of variance with post-hoc Bonferroni’s multiple comparisons test. NS: *p* > 0.05, *p < 0.05.

Cu-Li-nHA had the advantage of cell compatibility in that fibroblast-like BMSCs adhered to the pore wall (Figure 2E). The number of migrated cells in the Cu-Li-nHA group was the highest (*p* < 0.05), but there was no significant difference between the Cu-Li-nHA and the SDF-1 groups (*p* > 0.05) (Figures 3A,B). The number of migrated BMSCs in the nHA and Li-nHA groups was clearly less than that in the SDF-1 group (*p* < 0.05). Alkaline phosphatase expression in the Cu-Li-nHA group was significantly higher than that in the nHA and Li-nHA groups. The number of calcium deposits was also visibly higher in the Cu-Li-nHA group than in the nHA and Li-nHA groups (Figures 4A,B). As shown in Figures 4C,D, the number of vascular-like structures in the Cu-Li-nHA group was the highest among the three scaffold groups (*p* < 0.05), but less than that in the positive group (*p* < 0.05). The expression levels of Runx2, HIF-1α, VEGF, and SDF-1 in BMSCs were significantly higher in the Cu-Li-nHA group than in the other groups (Figures 5A,B).

### Cu-Li-nHA Modulated BMSCs Homing for Treating ONFH *in Vivo*

**FIGURE 6:**
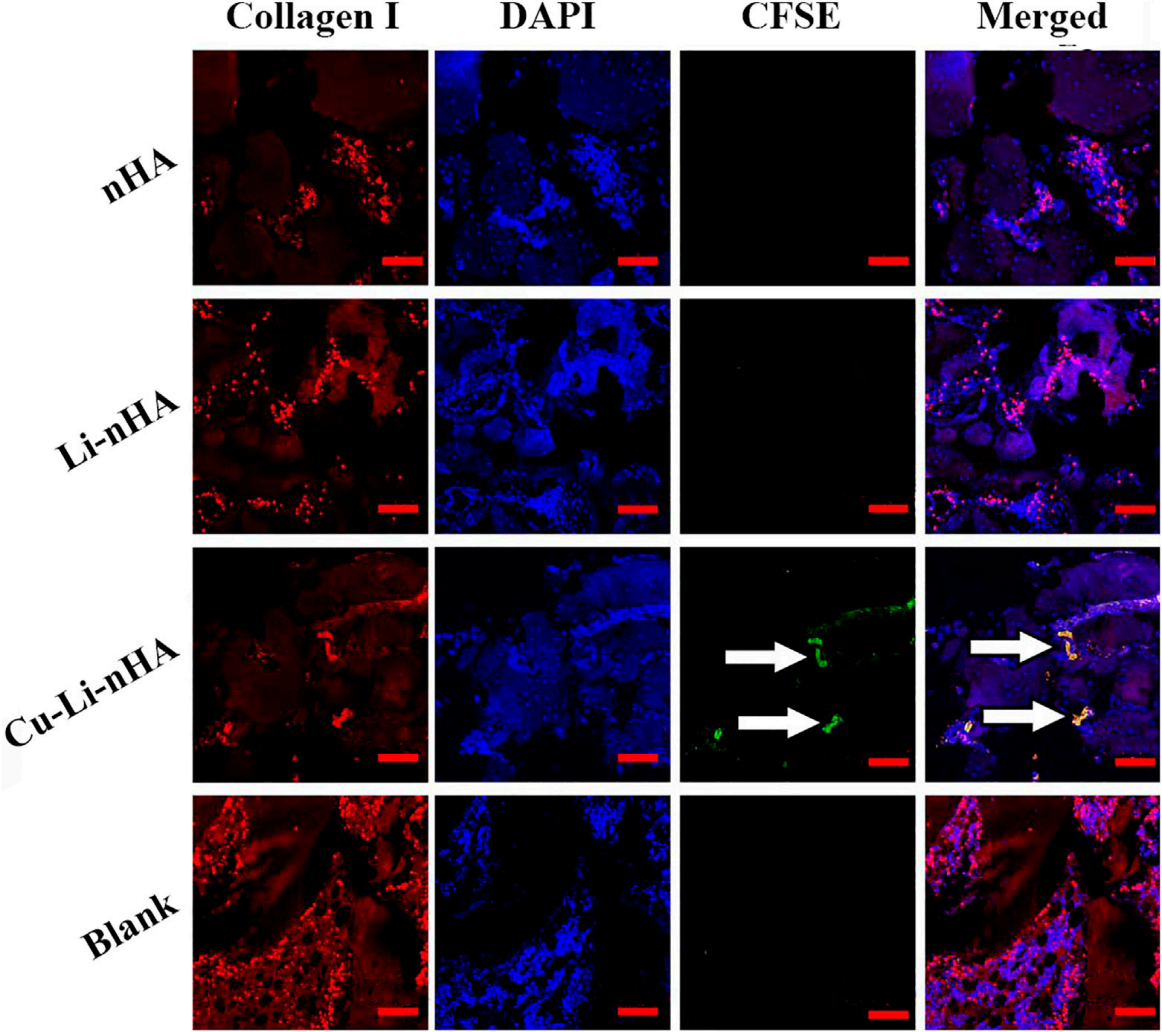
Immunofluorescence assays to verify BMSCs homing 2 weeks after injecting with exogenous CFSE-labeled BMSCs. BMSCs in the implanted regions were shown under the Collagen I signal (red), and homing BMSCs were shown under the CFSE signal (green). White arrow: CFSE-labeled BMSCs. Scale bar in the bottom right-hand corner of each image (red): 200 μm.

**FIGURE 7:**
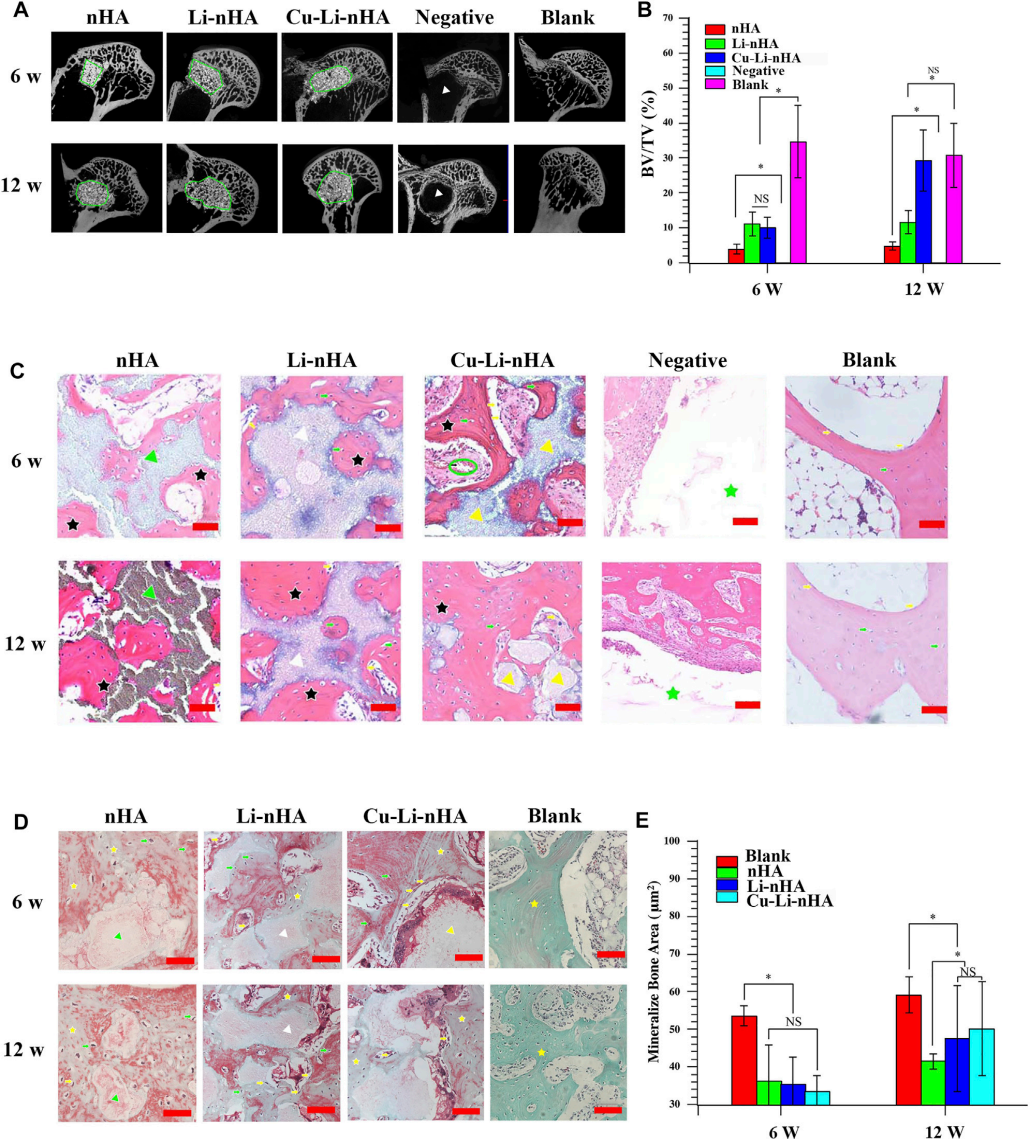
**(A,B)** Micro-CT observation and BV/TV values in the bone defect area. The regions within the green circle were implanted with scaffolds. Materials showed high density and new trabecular bone showed medium density. Negative Group: only drilling tunnels. Blank Group: without ONFH modeling and scaffold-implantation operation. White triangle: region of drilling. **(C)** HE staining. Black asterisk: trabecular bone. Green asterisk: drilling region. Yellow arrow: lining cell. Green arrow: osteocyte. Green triangle: nHA scaffold. White triangle: Li-nHA scaffold. Yellow triangle: Cu-Li-nHA scaffold. Green circle: neovascularization. Negative Group: only drilling tunnels. Blank Group: without ONFH modeling and scaffold-implantation operation. Scale bar in the bottom right-hand corner of each image (red): 100 μm. **(D,E)** Goldner staining and mineralize bone area analysis. Mineralized bone was stained in green, and osteoid was in red. Yellow asterisk: mineralized trabecular bone. Yellow arrow: lining cell. Green arrow: osteocyte. Green triangle: nHA scaffold. White triangle: Li-nHA scaffold. Yellow triangle: Cu-Li-nHA scaffold. Blank Group: without ONFH modeling and scaffold-implantation operation. Scale bar in the bottom right-hand corner of each image (red): 50 μm. Statistical analysis was conducted using a one-way analysis of variance with post-hoc Bonferroni’s multiple comparisons test. NS: *p* > 0.05, *p < 0.05.

Cells *in situ* were observed in all groups, whereas CFSE-labeled cells were observed only in the Cu-Li-nHA group (Figure 6). The materials showed high density in the bone defect area under micro-CT, whereas the new trabecular bone showed medium density (Figure 7A). A greater medium-density area could be observed in the Li-nHA and Cu-Li-nHA groups than in the nHA group after 6 weeks, and the density of the implanted region in the Cu-Li-nHA group was close to that in the blank group after 12 weeks. BV/TV showed similar trends after 6 (Cu-Li-nHA vs. nHA, *p* < 0.05) and 12 weeks (Cu-Li-nHA vs. Li-nHA, *p* < 0.05) (Figure 7B).

**FIGURE 8:**
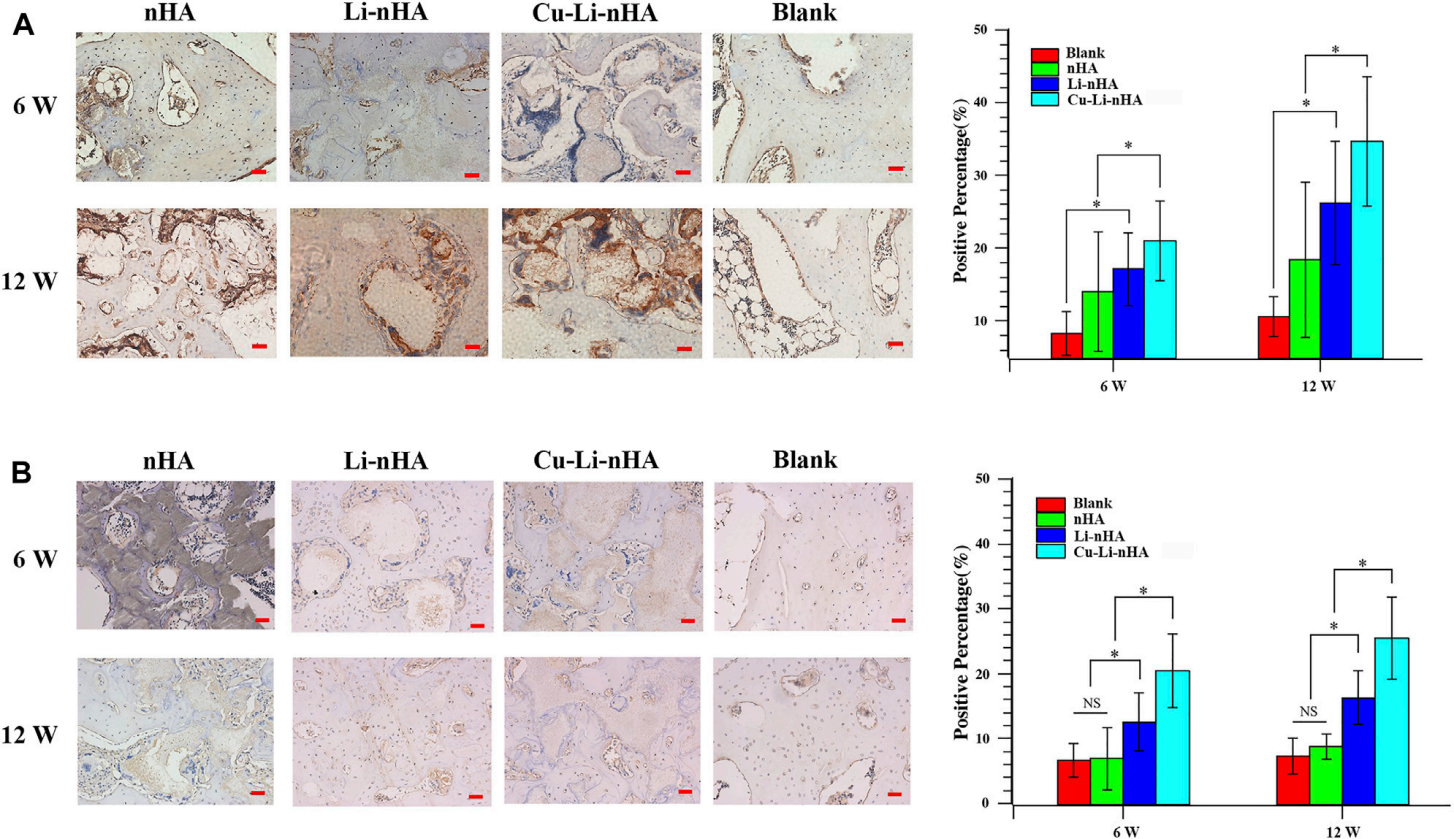
Immunohistochemistry and positive ratio analysis of OCN **(A)**, Runx2 **(B)**. Blank Group: without ONFH modeling and scaffold-implantation operation. Scale bar in the bottom right-hand corner of each image (red): 100 μm. Statistical analysis was conducted using a one-way analysis of variance with post-hoc Bonferroni’s multiple comparisons test. NS: *p* > 0.05, *p < 0.05.

**FIGURE 9:**
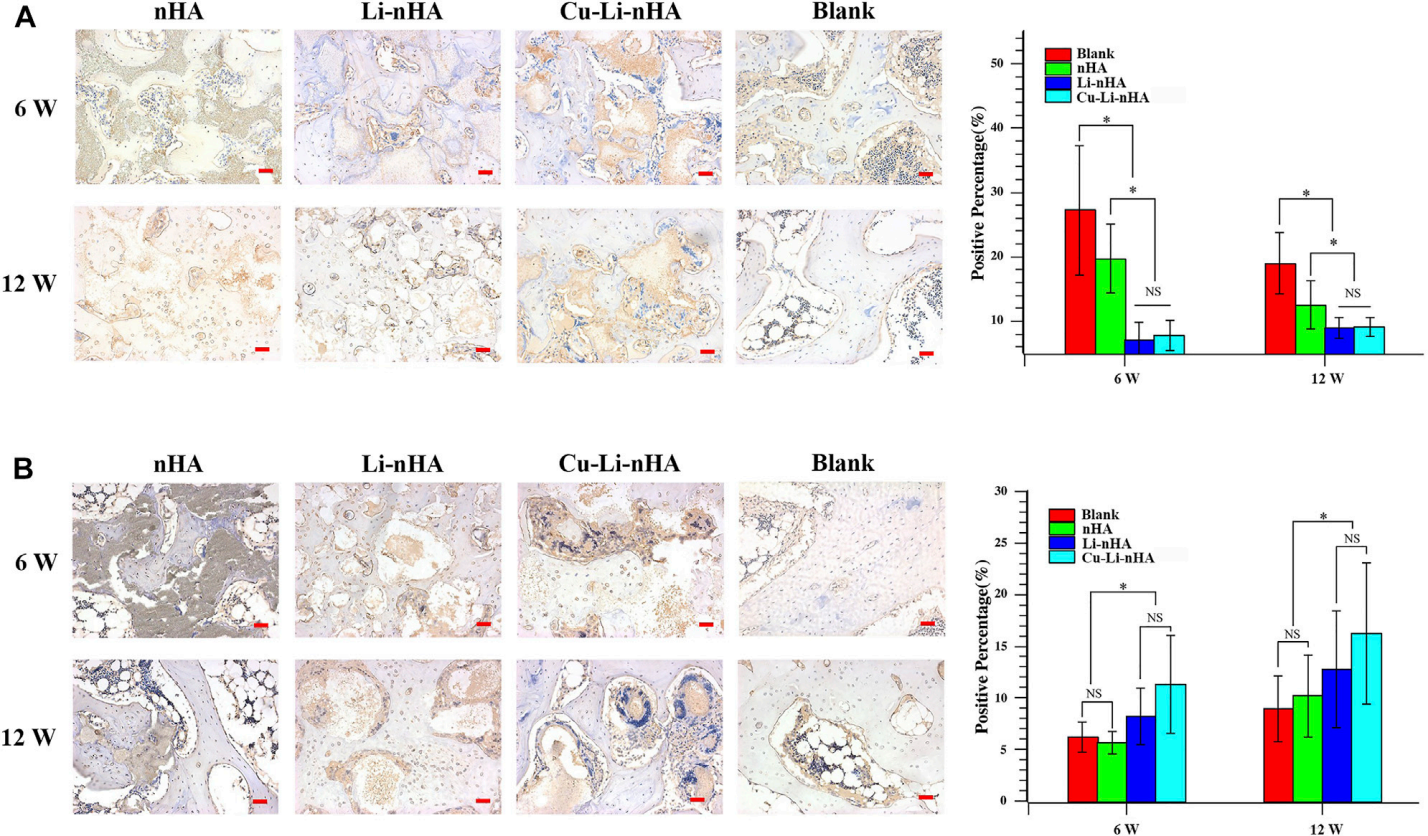
Immunohistochemistry and positive ratio analysis of GSK-3β **(A)**, β-catenin **(B)**. Blank Group: without ONFH modeling and scaffold-implantation operation. Scale bar in the bottom right-hand corner of each image (red): 100 μm. Statistical analysis was conducted using a one-way analysis of variance with post-hoc Bonferroni’s multiple comparisons test. NS: *p* > 0.05, *p < 0.05.

**FIGURE 10:**
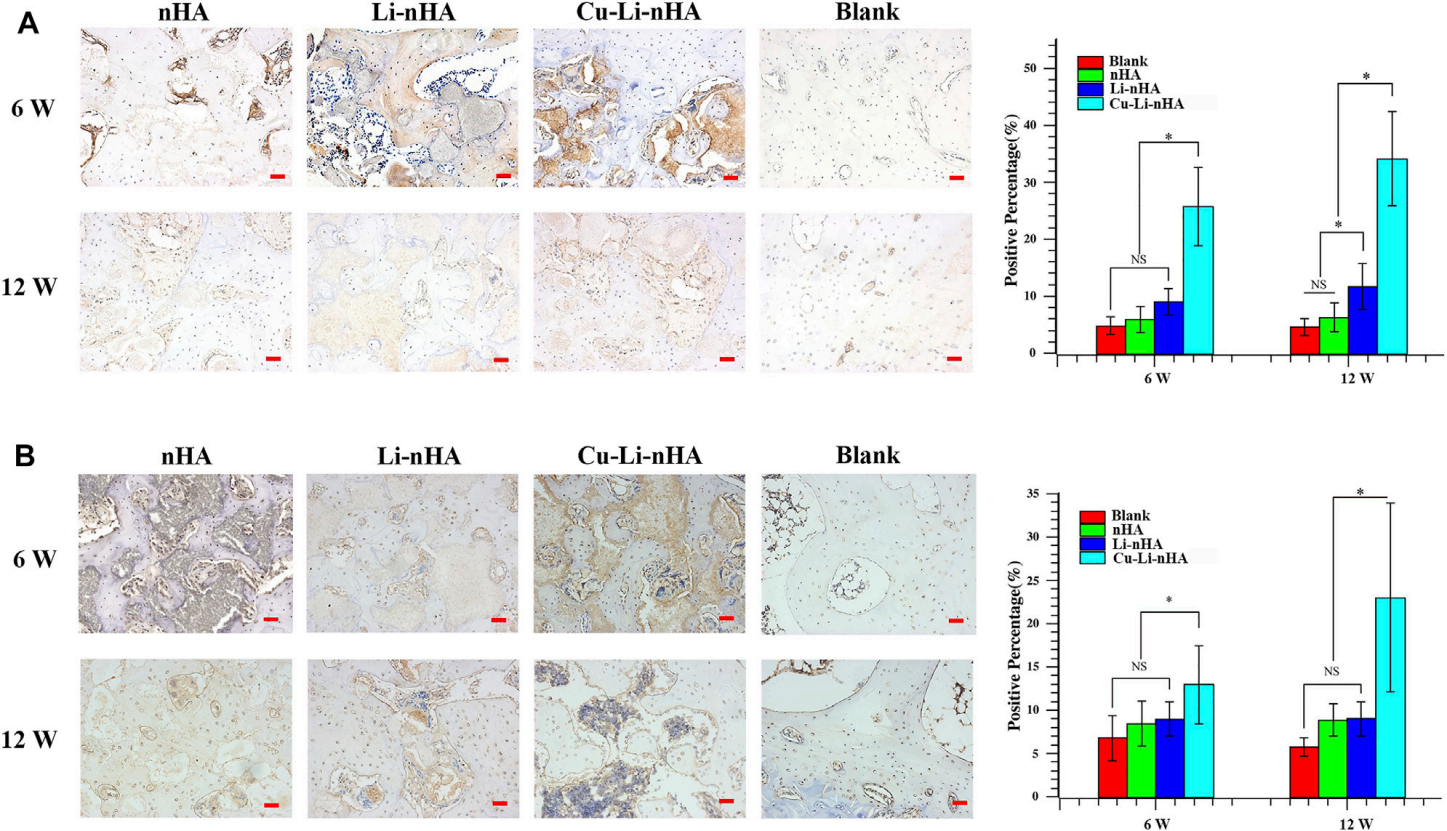
Immunohistochemistry and positive ratio analysis of VEGF **(A)**, SDF-1 **(B)**. Blank Group: without ONFH modeling and scaffold-implantation operation. Scale bar in the bottom right-hand corner of each image (red): 100 μm. Statistical analysis was conducted using a one-way analysis of variance with post-hoc Bonferroni’s multiple comparisons test. NS: *p* > 0.05, *p < 0.05.

HE staining showed new trabecular bone formation in the nHA, Li-nHA, and Cu-Li-nHA groups, and the shape of the trabecular bone in the Cu-Li-nHA group was intact. Abundant neovascularization was also observed in the Cu-Li-nHA group (Figure 7C). Goldner staining showed that trabecular bone mineralization in the Cu-Li-nHA and Li-nHA groups was higher than that in the nHA group (*p* < 0.05), but lower than that in the blank group (without ONFH modeling and scaffold-implantation operation) (*p* < 0.05) (Figures 7D,E). The expression levels of OCN, Runx2, β-catenin, VEGF, and SDF-1 in the Cu-Li-nHA group were higher than those in the other groups (*p* < 0.05) (Figures 8, 9, 10). In addition, the expression levels of GSK-3β in the Cu-Li-nHA and Li-nHA groups were significantly lower (*p* < 0.05).

## Discussion

In current study, we evaluated the Cu-Li-nHA porous composite scaffold for GCs-ONFH repair and we interestingly found that Cu-Li-nHA with biocompatibility and osteoconductivity, enabled MSCs recruitment to the target region and induced osteogenesis. The results indicated that Cu-Li-nHA promoted MSCs homing and enhanced bone regeneration in ONFH.

Surgical reconstruction usually requires tissue transplantation to restore normal structure and function (Zhao et al., 2021). However, autografts and allografts both present certain issues problems, such as additional surgical procedures and immune rejection (Dimitriou et al., 2011). Repair for ONFH is also faced with such challenges. To overcome these limitations, *in situ* tissue regeneration technology has been designed, which utilizes the implantation of bioactive scaffolds to recruit host progenitor cells, and simultaneously provides optimal microenvironment, contributing to proliferation and differentiation of recruited cells, finally promoting tissue regeneration (Lutolf et al., 2009; Ko et al., 2013). Several studies have confirmed the superiority of MSCs homing in improving the quantity and efficacy of regeneration of different tissues (Karp and Leng Teo, 2009; Xiang et al., 2020; Chen et al., 2021). For instance, Wang succeeded in BMSCs recruitment and osteogenic differentiation by loading hydroxyapatite/polyacrylonitrile scaffolds with SDF-1 (Wang et al., 2019). Moreover, patients with GCs-ONFH present a decreased number of BMSCs in the lesion area (Hernigou et al., 1999). Chronic abuse of GCs results in the differentiation of BMSCs into adipose tissue and cartilage, thereby reducing the reserve of stem cells (Cui et al., 1997; Houdek et al., 2016). Thus, a well-designed material must be fabricated to recruit MSCs and provide an osteogenic microenvironment for GCs-ONFH reconstruction.

SDF-1 is one of the factors that induce the directional migration of cells (Lau and Wang, 2011). Scaffolds composite with SDF-1 are efficient in repairing organ defects by stimulating BMSCs homing (Chen et al., 2015; Chen et al., 2019). SDF-1 in local tissues not only mobilizes adjacent BMSCs, but also recruits MSCs from the peripheral blood (Ceradini and Gurtner, 2005). HIF-1α regulates SDF-1 expression, and theoretically, Cu also upregulates HIF-1α to increase SDF-1 levels indirectly. Chen found that Cu increased BMSCs motility and recruitment through Rnd3 pathway-dependent cytoskeletal remodeling (Chen et al., 2020). Hence, we designed a composite scaffold by doping Cu into Li-nHA to enhance tissue regeneration potential through the MSCs homing. We successfully developed a Cu-Li-nHA scaffold with a three-dimensional porous structure and good compressive strength. In an *in vitro* chemotaxis experiment, Cu-Li-nHA promoted the directional migration of BMSCs. Moreover, the effect of Cu-Li-nHA on chemotaxis was not inferior to that of SDF-1. Cu-Li-nHA also induced exogenous BMSCs homing to the scaffold site *in vivo* and highly expressed SDF-1. Thus, Cu-Li-nHA mobilized the intrinsic reserves of MSCs to repair the damaged region of ONFH.

Li-nHA has been reported to enhance osteogenic differentiation of BMSCs *via* the Wnt pathway (Li et al., 2018a; Luo et al., 2018). Our results are consistent with the findings of previous studies. In addition, the angiogenesis of Cu-Li-nHA was explored. Neovascularization increases local blood supply in osteonecrosis, which benefits new bone ingrowth for reconstruction (Zhao et al., 2016). VEGF plays an important role in this process. Kim found that VEGF-loaded biomaterials provided an appropriate environment for accelerated osteogenesis (Kim et al., 2021). Similarly, our study indicated that Cu doping in the composite scaffold also increased vascular-like structures and promoted trabecular bone formation, largely because Cu can activate VEGF by inhibiting the degradation of HIF-1α (Feng et al., 2009). Cu-Li-nHA improved the microcirculation of ONFH to create more conducive conditions for MSCs differentiation.

This study also has several limitations. First, we did not include a Cu-nHA group in the study. Previous studies have demonstrated that Li-nHA has osteogenic properties but Li with limited ability of cell homing (Li et al., 2018a). Therefore, we doped Cu into Li-nHA to improve tissue regeneration potential *via* Cu modulating MSCs homing for promoting the repair of ONFH, instead of verifying role of Cu in MSCs mobilization alone. Second, we had not set relevant experiments by regulating HIF-1α/SDF-1 pathway for further validation. Third, further research should be conducted using other animal models of ONFH to confirm the reconstruction effect of Cu-Li-nHA.

The current study demonstrated that the Cu-Li-nHA composite scaffold could induce MSCs homing and improve osteogenesis and angiogenesis, consequently promoting GCs-ONFH repair. Thus, Cu-Li-nHA implantation may serve as a potential therapeutic strategy for ONFH in the future.

## Data Availability

The original contributions presented in the study are included in the article/Supplementary Material, further inquiries can be directed to the corresponding author.
