# Supplementary material for: Copper-Lithium-Doped Nanohydroxyapatite Modulates Mesenchymal Stem Cells Homing to Treat Glucocorticoids-Related Osteonecrosis of the Femoral Head
Source: Front Bioeng Biotechnol. 2022 Jun 2;10:916562. doi: 10.3389/fbioe.2022.916562 (PMC9201282; doi:10.3389/fbioe.2022.916562)
Supplement: Supplementary file 1 [file DataSheet1.DOCX]

**Supplementary material**

**Culture and identification of BMSCs**

BMSCs were taken from the bone marrow of suckling New Zealand white rabbits under aseptic conditions. Femurs and humeri were rinsed with phosphate buffered saline (PBS, GIBCO, USA) and low glucose Dulbecco’s modified Eagle’s medium (LG-DMEM, GIBCO, USA). BMSCs were collected by flushing medullary cavities slowly with LG-DMEM containing 10% fetal bovine serum (GIBCO, USA) and then cultured in a 37°C, 5% CO_2_ incubator. When the cell fusion rate was 80–90%, BMSCs were digested with 0.25% trypsin (Gibco, USA) and passaged at 1:2. The morphology of the cells was observed by inverted phase contrast microscopy (Olympus, Japan) at 24 h, 72 h, 10 d, and the third generation (Supplementary figure 1). The purity of BMSCs was tested by flow cytometry (Becton, Dickinson and Company, USA) (Supplementary figure 2).


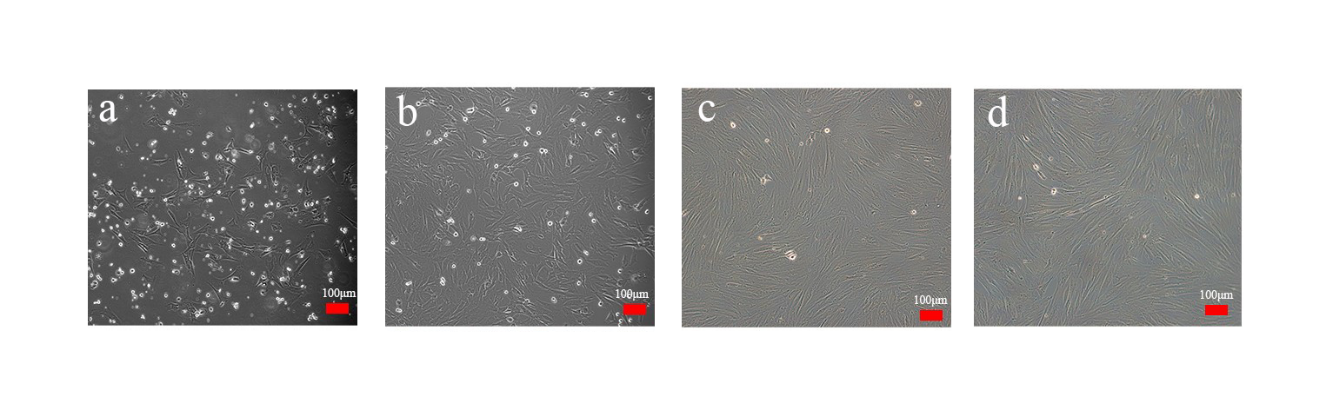


**Supplementary figure 1** Observation of the BMSCs’ morphology at (a) 24 h, (b) 72 h, (c) 10 d and (d) the third generation. Scale bar: 100 μm.


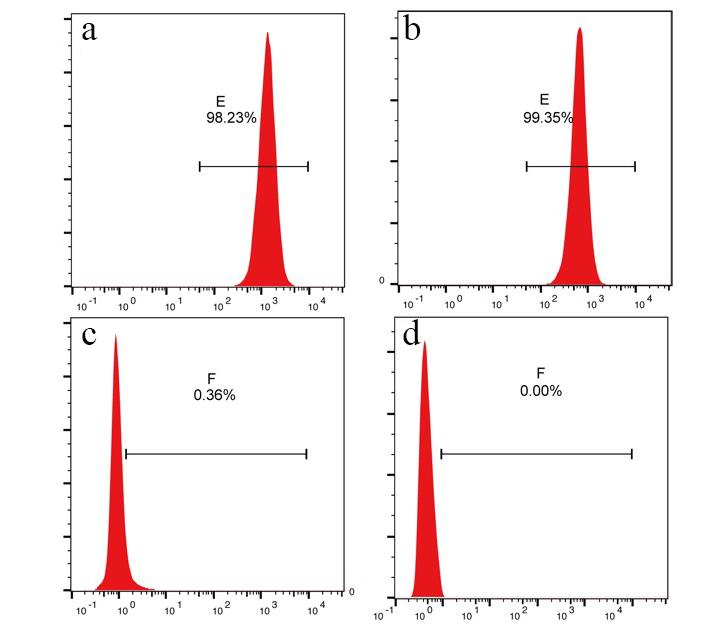


**Supplementary figure 2** BMSCs’ identification by flow cytometry. Positive rate: (a) CD29-FITC 98.23%, (b) CD44-FITC 99.35%, (c) CD34-FITC 0.36%, (d) no fluorescent signal 0.00%.

**Incidence of ONFH in rabbits**

In order to confirm the success rate of our ONFH model, twenty of rabbits were randomly divided into the ONFH group and the control group. HE staining was performed four weeks after the last injection of methylprednisolone (Supplementary figure 3). The rate of empty bone lacuna was significantly higher in the ONFH group (74.83±10.45) than in the control group (9.42±2.13) (*p* < 0.05).


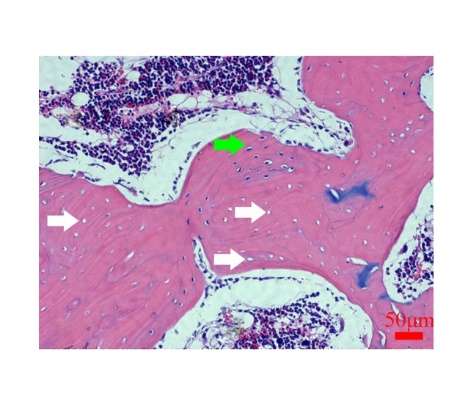


**Supplementary figure 3** HE staining of ONFH model. White arrow: empty bone lacuna. Green arrow: normal osteocytes. Scale bar: 50 μm.

**Figures of the materials implantations**

**
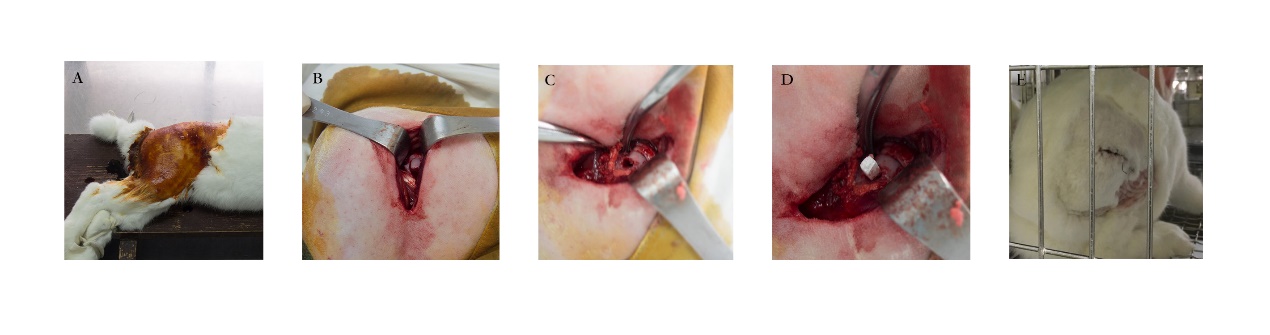
Supplementary figure 4** Materials implantations process. (A) Disinfection. (B) Femoral head exposed. (C) Drilling. (D) Materials implantation. (E) The incision healed well two weeks after surgery.


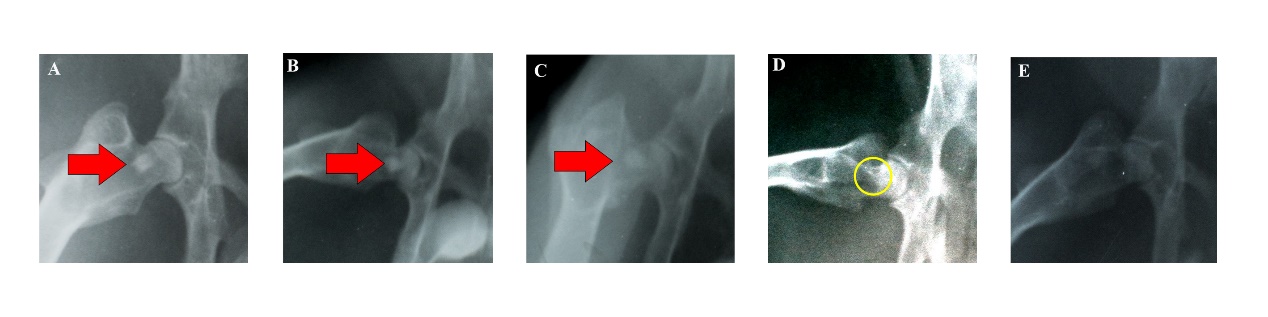


**Supplementary figure 5** X-ray two weeks after surgery. (A) nHA group. (B) Li-nHA group. (C) Cu-Li-nHA group. (D) Negative group. (E) Blank group. Red arrow: implanted material. Yellow circle: drilling position.
